# Gender disparity as a threat to the mental well-being of young Sri Lankan women

**DOI:** 10.1192/bji.2018.29

**Published:** 2019-11

**Authors:** Miyuru Chandradasa, Layani Champika Rathnayake

**Affiliations:** 1 Lecturer, Department of Psychiatry, Faculty of Medicine, University of Kelaniya, Sri Lanka. Email: miyuruc@kln.ac.lk; 2 Honorary Senior Lecturer, Faculty of Medicine, Nursing and Health Sciences, Monash University, Australia; 3 Specialist Registrar, Latrobe Regional Hospital, Australia

**Keywords:** Mental health, women, Asia, Sri Lanka, gender

## Abstract

Sri Lanka ranks highest in the region for human development. Despite producing the first female head of state in the world, the country has failed to achieve substantial gains in the gender inequality indices in the past decade. In recent years, the proportion of females in secondary education has equalled that of males, and young women have become the majority among the university entrants. These educated young women are likely to face psychosocial distress in a patriarchal society where they would be expected to fulfil traditional gender roles. This article describes gender disparities that could affect the mental well-being of young Sri Lankan women and the need for awareness among mental health professionals in the country.

In January 2018, Sri Lanka made international headlines due to the removal of an archaic law that barred women from purchasing alcohol (BBC, 2018). Rights activists praised this as a positive change that recognised the rights of women as equals. However, the government reinstated the law after opposition from several quarters who claimed that women are more vulnerable to alcohol-related medical complications and were concerned about their social responsibilities as mothers. Women who stood up against this reinstatement were mocked on social media networks. This ideological conflict surfaced the decades-old gender inequalities in the post-colonial society. Here we describe the gender disparities that could affect the mental well-being of young Sri Lankan women of today and tomorrow.

## Human development

Europeans ruled parts of Sri Lanka from 1505 until the independence from the British Empire in 1948. The country suffered a brutal armed conflict from the early 80s until 2009 which started as an ethnic conflict between the majority Sinhalese and minority Tamils. This later expanded into a war between the Liberation Tigers of Tamil Elam, a separatist militant organisation with international terrorist affiliations, and the Sri Lankan Government (Stanford University, 2015). Since the end of the war, the country has progressed to the high human development (HDI) category, as classified by the United Nations Development Programme (UNDP, 2016). The parameters considered include life expectancy at birth, mean years of schooling and gross national income per capita.

## Gender inequality

In contrast to the HDI, the country has failed to progress in the Gender Inequality Index. Sri Lanka is in the 87th position out of 155 countries, but it was in the 75th position out of 148 in 2012 (UNDP, 2013, 2016). Gender inequality is generally assessed by the maternal mortality rate, adolescent birth rate, proportion of females in parliament, secondary education and workforce participation. Sri Lanka has a far better maternal mortality ratio (30 per 100 000 live births) and adolescent birth rate (14.8 per 1000 women) compared to the regional figures for South Asia. However, the proportion of women in parliament is 4.9%, which is far below the regional figure of 17.4%. Sri Lanka ranks highly in terms of secondary education, with 80.2% of females reaching this academic level; South Asian figures are at 36.9%. Finally, Sri Lankan women make up only 30.2% of the workforce, which is on par with regional figures (UNDP, 2016). Even though the degree of gender inequality is less than in neighbouring countries, many Sri Lankan women tend to be engaged in traditionally feminine occupations and earn less than men even in comparable positions. In addition, there is a high concentration of females in low-level positions and males in high-level positions (Gunawardane, 2016). Sri Lankan women play a major role in the agricultural industry, but their labour is valued less than that of men (Premarathna, 2018).

## Women and politics

Sri Lankan women obtained voting rights in 1931, just 3 years after women in the UK. Furthermore, when Sirimavo Bandaranaike was elected prime minister in 1960, Sri Lanka was the first country in the modern world to have a female head of state (Kearney, 1981). Despite having a low percentage of female parliamentarians, women have played a significant role in the country's post-colonial political culture. In Sri Lanka's documented history spanning 2600 years, women have held high social and political responsibility. Even today, women are considered the core of Sri Lanka's moral identity (Lynch, 2007).

## Poverty and women

Currently poverty is the main contributor of psychosocial vulnerability in adolescent girls in post-conflict settings in northeastern parts of the country (Samuels *et al*, 2017). In the 1990s, many men were at the war front and the government had to create employment opportunities for young women (Lynch, 2007). Many apparel factories were established in free-trade zones and this industry has remained the country's largest net foreign exchange earner since 1992 (Dheerasinghe, 2009). Despite families valuing education, a significant number of young women dropped out from secondary education due to poverty, romantic relationships or early marriage (Samuels *et al*, 2017). Many in this group ended up as garment workers living away from their traditional social networks. These young women are overworked, underpaid and subjected to abuse, highlighting the fact that increased numbers of women in the workforce does not necessarily translate to improved gender equality (Lynch, 2007).

## Gender and culture

Young women in Sri Lanka experience stigma associated with gender-based cultural norms. This plays a substantial role when adolescent girls access mental health services. Parents would insist on speaking on behalf of the young person in the context of overprotective parenting in a collectivistic society (Freeman, 1997). Most of these gender-based norms are unrelated to the majority religion, Theravada Buddhism. In fact, Buddhism is known to promote gender equality, where Buddha taught that both men and women could purify their minds and achieve enlightenment to become arahat. The female arahat monk Sanghamitta played a significant role in introducing Buddhism to Sri Lanka in the third century BC (Bartholomeusz, 1994). In addition, predominantly Buddhist countries have better gender equality compared with those favouring other traditional religions such as Hinduism and Islam (Prewitt-Freilino *et al*, 2012).

## Women and sexuality

Premarital sex is culturally not accepted, but more than 10% of school children and more than 20% of out of school adolescents are found to be sexually active (Thalagala *et al*, 2004). Additionally, the use of barrier contraceptives is low in the country compared with others (Premadasa *et al*, 2016). This has potentially made young women vulnerable to sexually transmitted diseases and unwanted pregnancies. Abortion is a criminal offence in Sri Lanka unless performed to save the life of the mother. Therefore, young women who are pregnant due to premarital sex would face substantial social pressure to seek illegal and unsafe abortions by unqualified practitioners, leading to physical and psychological trauma (Abeyesekera, 1997).

Unlike many other Asian nations, the Sri Lankan population does not show a significant preference to sons, probably due to absent disadvantages for females in health and education (Fikree & Pasha, 2004). Studies have found more than a third of women had experienced intimate partner violence in their lifetimes and young women are more vulnerable. In contrast, Sri Lanka has fewer issues relating to dowries and child marriages, which is a positive difference to other Asian nations (Jayasuriya *et al*, 2011). In 2005, specific legislation was made against domestic violence which was a landmark for women's rights. Unfortunately, marital rape is not a criminal offence unless the spouse is judicially separated (Jayatilleke *et al*, 2010). Furthermore, young Sri Lankan women are known to be harassed and discriminated against due to their clothing and a recent survey by the United Nations Population Fund found that 90% of women using public transport reported being sexually harassed (UNFPA, 2017). Motherhood is considered an essential component of a woman's social identity. In Sri Lankan couples suffering from infertility, pressure from the extended family, myths about the responsibility of infertility and poor marital communication could lead to significant psychological distress in young women (Lansakara *et al*, 2011).

## The next generation of women

Although an equal proportion of men and women obtain secondary education, there are higher numbers of women in tertiary education. In the 2015/2016 academic year, 63% of all state university entrants were females; women made up a majority in undergraduate medicine (59%) and law (86%) (UGC, 2016). This has been the trend for the past few years, and a significant majority of the graduates are now females, making it appear that women are more educated than men. The educational capabilities and employment of Sri Lankan women influence their power and control in their families (Malhotra & Mather, 1997).

## Mental health of women

The female suicide rate in Sri Lanka is one of the highest in the world and there are high rates of self-harm in young women living in rural, disadvantaged groups. These acts are mostly made impulsively against perceived wrongful treatment, often related to family disputes and conflicts with intimate partners (Marecek, 2006). In the aftermath of the 2004 tsunami, it was found that depressive psychopathology among adolescents was significantly associated with depression in their mothers. Furthermore, positive mother–child relations directly related to better adolescent psychological health (Wickrama & Kaspar, 2007). This is understandable in the Sri Lankan context where family cohesiveness is highly regarded and motherhood is valued and attributed with serious responsibility, which is highly beneficial to the children. However, it could be challenging for a young women to fulfil the social role of a traditional mother when they are more likely to be educated and employed than the women of the past, who were mostly homemakers. This role change of the Sri Lankan mother could induce rifts between married partners and in-laws, where grandparents are expected to be significantly involved in childrearing. This could be an issue for the grandparents because, in this Buddhist-majority country, elderly people engage in deeply religious activities in their preparation for a noble birth after their death, and temples are flocked by senior citizens learning and practising meditation.

## The future

It appears that young Sri Lankan women of the future will be more educated than men. However, they are likely to face a changing patriarchal society and are likely to develop psychological issues that would require greater support from health professionals. The mental health services tackle a heavy load of major psychiatric disorders and the subspecialties are not well developed (Chandradasa & Champika, 2018). Nevertheless, with the end of armed conflict and improved living conditions, a substantial proportion of Sri Lankan psychiatrists who trained in high-income countries are returning to their home country. This foreign exposure is a compulsory advanced training component of the Sri Lankan postgraduate psychiatry programme (Chandradasa & Kuruppuarachchi, 2017). Their experience in more gender-sensitive services in high-income countries such as the UK and Australia as well as their local training could be employed to develop more structured and culturally appropriate mental health services for Sri Lankan women. Collaborative work between mental health specialists, professional organisations, health administrators and policymakers is essential to make this a reality.
